# Caregiver dementia training in caregiver‐patient dyads: Process evaluation of a randomized controlled study

**DOI:** 10.1002/gps.5404

**Published:** 2020-09-17

**Authors:** Elizabeth G. Birkenhäger‐Gillesse, Wilco P. Achterberg, Sarah I. M. Janus, Sytse U. Zuidema

**Affiliations:** ^1^ Department of General Practice and Elderly Care Medicine University of Groningen, University Medical Center Groningen Groningen The Netherlands; ^2^ Laurens Care Centers, Division Long Stay Rotterdam The Netherlands; ^3^ Department of Public Health and Primary Care Leiden University Medical Center Leiden The Netherlands

**Keywords:** caregiver, dementia, psychosocial intervention, training

## Abstract

**Objectives:**

We performed a randomized controlled study to evaluate the effects of caregiver training on the well‐being of both people with dementia and their caregivers. Before the effect analysis, we conducted a process evaluation to estimate internal and external validity. This was anticipated to augment our understanding of the outcomes.

**Methods:**

We focused on three questions. (a) Was the intervention performed as planned (internal validity)? (b) Can qualitative data be used to inform how the intervention evoked change? (c) Can the study outcomes be extrapolated to all caregivers living with people who have dementia (external validity)?

**Results:**

Responses from participants assigned to the intervention group suggested that the intervention was feasible, could be performed as planned, and that modelling and discussions between participants were important. However, participant recruitment to the entire study was ultimately laborious because participants had issues with the study design (risk of being assigned to the control group) and referrers lacked familiarity with the training (new type of intervention). Participants were also younger and better educated compared with the general population. Some dropouts in the follow‐up period occurred due to the number of questionnaires, and this was more pronounced in the control group.

**Conclusions:**

Although we achieved high internal validity, we lack certainty about the external validity. We not only experienced general difficulty in recruiting participants but also tended to recruit a biased sample that was relatively young and well educated. These factors combine to limit our ability to extrapolate the results to the general population.

**Key points**

- Internal validity is mainly dependent on the organizational quality of the study, and therefore, can be controlled.
- The external validity of a study with participants in the population is dependent on many factors, and these can only partially be controlled.
- Qualitative data are important because they help with our understanding of quantitative data analyses and the way change is affected.

## 1 OBJECTIVE

Most people with dementia (PWD) live in the community,^1^ and Dutch demographic data indicate that about 35% of these share a household with a spouse or partner who provides care.^2^ However, dementia is associated with behavioral problems and dependency, which can lead to high and persistent rates of caregiver burden^3^, ^4^ that may ultimately lead to depression and early nursing home placement.^4^, ^5^

The burdens placed on caregivers can be exacerbated by social isolation, lack of knowledge, poor skills, inadequate coping, guilt and a poor marital relationship. By contrast, protective factors include the presence of practical and emotional support and the use of problem‐focused coping.^6^ Caregiver interventions typically offer psychological or educational elements, help to develop caregiver support systems, or combine these in some way. However, the heterogeneity of available interventions and the outcomes that are measured make it difficult to identify the most effective and relevant interventions.

To date, most reviews have only indicated that caregiver programs have mild to moderate effectiveness compared with care as usual. In subgroup analyses, stronger positive effects have been seen on caregiver burden, psychological distress and caregiver knowledge.^7^, ^8^, ^9^, ^10^, ^11^ Examples of the characteristics of effective interventions in these studies are group training, social support, cognitive therapy, intensive support, multiple components (ie, in multi‐model programs), focus on the caregiver/patient dyad, caregiver training and psychological education.

We previously conducted a randomized controlled study to estimate the effect of an intensive multicomponent caregiver training program that comprised psychological, educational and social elements.^12^ Concomitantly with the caregiver training, partners followed a program that focused on helping them to deal with the changes that come with dementia. We based this training on the “Going To Stay at Home” intervention developed in Australia^6^, ^13^ and named it “More at Home with Dementia” (in Dutch: *Beter Thuis met Dementie*). Our understanding of the outcomes was augmented by conducting a process evaluation before the effect analysis, which allowed us to estimate the internal and external validity. Internal validity concerns the quality of the intervention itself, whereas external validity concerns the quality of sampling and the total study population. Further, the process evaluation can help when interpreting discrepancies between expected and observed outcomes.^14^

## 2 METHODS

### 2.1 Study design and planning

Detailed information on the methods and content of the randomized controlled “More at Home with Dementia” study is presented elsewhere.^12^ To avoid bias when interpreting the data, we conducted the process evaluation before final processing.

### 2.2 Intervention: setting and program components

The intervention lasted a total of 5 days and took place in a holiday accommodation providing bedrooms for six couples. Groups comprised two to six participant dyads, and during their stays, caregivers attended fourteen psycho‐educational sessions. These were delivered in an informal setting by specialist staff that included a psychologist, a physiotherapist, an occupational therapist, an elderly care physician, a speech therapist, a dietician and a social worker. Sessions included didactic elements, group work, modelling and role play. A facilitator's guide was used to improve consistency between workshops. During this training, the PWD engaged in general pleasant activities and sessions that focused on coping with dementia. Each couple was asked to contribute 100 euros for the 5‐day course, which equates to about 2.7% of an average monthly gross income in the Netherlands.^15^

### 2.3 Recruiting participants and follow‐up

Participants were recruited by either professional referral or self‐referral. In the Netherlands, dementia is diagnosed by geriatricians, neurologists or general practitioners (GPs). Patients and their caregivers are then assigned a case manager and, if desired, are referred to a day care center. Therefore, the project was promoted among these groups, with each asked to refer appropriate participants. This helped us to focus on reaching the caregivers of PWD early in the course of dementia, whom we thought would benefit most from the intervention. Given that dementia case managers have multiple contacts with caregivers, we assumed that they would be important referrers. In addition, we undertook various activities to stimulate interest and participation in the general public (these are described in detail in the results). Participants in the control group were offered the option to attend a free program at a museum for PWD and their caregivers.

Quantitative data on study outcomes were collected at baseline and at 3 and 6 months after the intervention. Caregivers were asked to complete the following: Care‐related quality of life,^16^ self‐rated burden scale,^17^ objective caregiver burden, RAND‐36 SF experienced health,^18^ Euroqol‐5 Dimensions,^19^ CES‐depression,^20^ HADS‐anxiety^21^ and perseverance time.^22^ Outcomes for PWD (informant rated) were assessed using the Neuropsychiatric Inventory,^23^ KATZ‐15,^24^ Dementia Quality of Life (self‐rated when possible),^25^ and Cohen‐Mansfield Agitation Inventory.^26^ We also sought details of resource utilization and psychotropic drug use from both the caregiver and the PWD. Participants randomized to the control group received usual care.

### 2.4 Components of the process evaluation

There are many ways to conduct a process evaluation.^14^, ^27^, ^28^ Among these, the UK Medical Research Council provides a practical framework for evaluating public health interventions.^14^ Given that our study was not conducted within institutions, we considered this approach to be appropriate and focused on three key issues:

1. Was the intervention performed as planned? Here, we assessed the quality of the intervention and tested the internal validity.
2. Do the quantitative and qualitative data build on each other and provide information on how the intervention evoked change?
3. Can the study outcomes be extrapolated to all caregivers living with a PWD? Here, we assessed the quality of the sampling and the resulting study population to test the external validity.

### 2.5 Internal validity: quality of the intervention

To evaluate the intervention quality, we recorded the proportion of workshops delivered as planned and, when applicable, identified the reasons for divergence. Any changes in organization, together with the associated reasons, were also recorded. Participant adherence to the interventional components was assessed by analyzing the proportion of workshops attended, as well as the barriers and facilitators reportedly associated with attendance. The comments made by participants during follow‐up meetings after 3 and 6 months were used to evaluate the quality of the training and the caregiver's experience of the training.

### 2.6 External validity: recruitment and follow‐up

To estimate the external validity, we carefully scrutinized the recruitment and follow‐up procedures used in the intervention and control groups before and after randomization. We also estimated the proportion of couples who participated in the study when asked by a professional, and we compared the numbers of clinician‐referred to self‐referred participants. Finally, we monitored the follow‐up attrition rate, reasons for dropout, actual numbers of participants who completed the intervention per group, and the target number of participants.

### 2.7 Data collection for the process evaluation

To assess training completeness, we screened the timetables for each intervention week. A logbook was kept for each week to enable professionals to report details of the workshops they deemed relevant. These logbooks were screened for any deviations and for the attendance rates of participants. Summaries and recordings of feedback meetings were screened after 3‐ and 6‐months for comments on the training content and for information about emotional or other impacts of training. We then produced an overview of all recruitment activities. Case managers for dementia were asked to estimate the proportions of people receiving information on the training and agreeing to participate. The contact details of people who asked for information from the research assistant were collected, and if possible, reasons for nonparticipation were recorded. A research assistant systematically recorded the reasons why people stopped participating.

## 3 RESULTS

### 3.1 Internal validity: quality of the intervention

#### 3.1.1 Completeness of the intervention

Four couples participated in a pilot week before we started the full intervention, which then proceeded on sixteen occasions. To standardize the workshops, we organized for specialist staff to meet before the pilot week and after intervention weeks 10 and 14. Additionally, professionals were asked to give a plain language summary for the content of each workshop to be included in a syllabus that participants could use as a reference. Only 4% of the planned workshops had to be skipped due to logistical problems. An overview of the number of times workshops were performed, and by whom, is presented in Table 1.

**TABLE 1:** Summary of intervention completeness

Workshop	Professional	Number of times performed
Combating social isolation	Psychologist	16
Medical aspects of dementia	Elderly care physician	16
Planning for the future	Social worker	16
Re‐rolling	Psychologist	16
Reminiscence	Psychologist	12
Communication and swallowing problems	Speech therapist	16
Assertiveness	Psychologist	16
Therapeutic use of activities	Occupational therapist	16
Organization of work and safety in the home	Occupational therapist	16
Nursing skills	Registered nurse	16
Fitness	Physiotherapist	15
Nutrition	Dietician	16
Self‐care	Psychologist	16
Using community services	Social worker	12
Relaxation exercises^a^	Psychologist	16
Movement therapy^b^	Movement therapist	16
Music activity^a^		6

a Added as joint activity for the caregiver and person with dementia.

b Added to emphasize importance of activities and as a pleasant activity for the person with dementia.

#### 3.1.2 Changes in organization during the intervention

The first 8 weeks of the intervention took place in a holiday park. However, this location had some major disadvantages (eg, it was too expensive and had insufficient room for meetings) that led us to change to a bed and breakfast setting halfway through the study. Unfortunately, we could only rent this accommodation from Monday to Friday, so we had to deliver the 14 workshops over 4 rather than 5 days. This change was associated with no major issues, although participants did have less spare time.

#### 3.1.3 Intervention adherence by participants

During the 16 weeks, no participating couples left the interventions early. However, six workshops (2.7%) were skipped by participants for different reasons (eg, physical complaints of the caregiver or PWD or planned hospital visits).

#### 3.1.4 Participant feedback on the strengths and weaknesses of the organization

In total, 18 of the 24 couples who attended the holiday park and 21 of the 35 couples who stayed at the bed and breakfast attended feedback meetings at 3 and 6 months (18 were organized). Participants had little to say about the locations or logistical aspects, but nine who attended at the holiday park and five who attended at the bed and breakfast judged the intervention week as being too long and too intense. By contrast, other participants said they felt the experience was like a holiday because they were so well taken care of, did not have to think of everyday concerns and the group had a positive spirit (“a week of laughter and tears”). In five meetings, nine caregivers reported that their partners had not engaged in meaningful or suitable activities, but in three, the caregivers stated that their partners had experienced a fun week because of the activities and the company of other PWD. Participants in three meetings reported that they had noticed some overlap in workshop content.

### 3.2 Qualitative and quantitative information combined

At the 3‐ and 6‐month follow‐up meetings, participants were asked how they had experienced knowledge transfer. They indicated that the discussions during workshops had been very helpful, noting that conversations (often continuing after the workshops) helped them to benefit from the expertise of other participants. This was confirmed by our observation that participants often exchanged experiences and useful practical information during the meetings at 3 and 6 months. Participants told us the syllabus they took home was useful, with some commenting that it allowed them to read more about certain topics and to access the cited internet sites at their leisure. It also helped when they needed to inform people at home (eg, case managers or children) about what had been learnt, facilitating change in others.

### 3.3 External validity

#### 3.3.1 Activities focused on recruitment

A recruitment strategy was developed that involved a logo, a website, a short promotional film, a brochure, a Facebook page, an advertisement in a local newspaper and a regular newsletter distribution. We also gave presentations at all eight memory clinics and at fourteen local meetings of the Dutch Alzheimer Association in Rotterdam and surrounding areas. As appropriate, we visited or sent brochures to day care centers, welfare organizations, general practices and health centers, and meetings of dementia case managers. Information about the intervention was also posted in newsletters of relevant local and national organizations. Specifically, the Dutch Alzheimer Association placed information about the intervention on their website and on their Facebook page, and a popular local newspaper published an article that gave a positive impression.^29^ These were designed to spread the key message that it is important for caregivers to learn how to care for and live with a partner who has dementia. In addition, information was given about the organization and content of the workshops.

#### 3.3.2 Recruitment and participation rate

**FIGURE 1:**
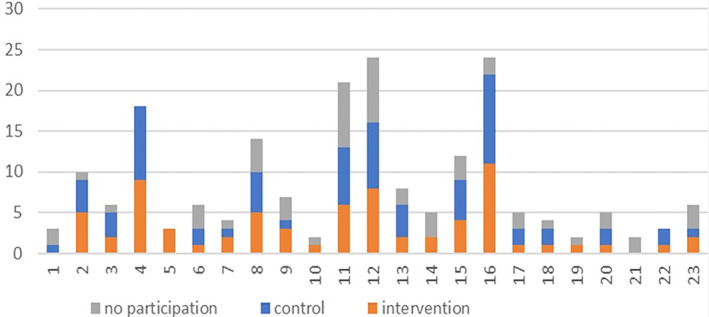
Relationship between recruitment activities and participant enrolment by recruitment activity. Participant numbers are shown in the vertical axis and study month is shown in the horizontal axis. Newsletter of the Dutch Alzheimer Association (month 4); advertisement local newspaper (month 7); Facebook Dutch Alzheimer Association (month 11); and news article local newspaper (month 16) [Colour figure can be viewed at wileyonlinelibrary.com]

The relationship between recruitment method and participant enrolment is shown in Figure 1. The participation rate increased considerably after publication of information by the Dutch Alzheimer Association and the article in a local newspaper, but the increases in enrolment rates were only temporary. The relationship between referral and participation is shown in Table 2.

**TABLE 2:** Participation by referral source

Referrer	Intervention group n (%)^a^	Control group n (%)^a^	Pilot n (%)^a^	Waiving after request for information n (%)^a^	Total n (%)^b^
Geriatrician	5 (33.3)	4 (26.7)	1 (6.7)	5 (33.3)	15 (7.5)
Case manager	28 (43.8)	20 (31.3)	2 (3.1)	14 (21.9)	64 (32)
General practitioner	22 (5)	1 (12.5)	1 (12.5)	4 (50)	8 (4)
Self‐referring	22 (33.3)	25 (37.9)	0	19 (28.8)	66 (33)
Unknown	14 (29.8)	21 (44.7)	0	12 (25.5)	47 (23.5)
Total	71	71	4	54	200

a Proportion of participants of total “referred by.”

b Proportion of total of requests for information.

Of the 200 people who contacted the organization by e‐mail or telephone for more information on the project, many (33%) reported that they had self‐referred after receiving information (either directly or via their children) from the internet, a newspaper, local Alzheimer meetings or other participants. Case managers referred a similar number of patients (32%), despite the advanced stages of dementia among their patients. Only a few were referred by a geriatrician (7.5%) or GP (4%). No information about recruitment was available in 23% of cases.

#### 3.3.3 Follow‐up

We included 142 of the 144 potential participants. After randomization, people in the intervention and control groups were sent questionnaires by post. Questionnaires that required additional instructions were excluded, such as the Neuropsychiatric Inventory, the Cohen‐Mansfield Agitation Inventory, the Dementia Quality of Life scale and the Global Deterioration Scale. Participants were given assistance to complete the questionnaires during the intervention week or during home visits by the research assistant (ie, at baseline). After 3 and 6 months, the caregivers in the intervention group who attended meetings (75% and 60%, respectively) received assistance from a research assistant to complete the questionnaires. Participants in the control and intervention groups who did not attend meetings were sent questionnaires by post and assisted by telephone if requested.

#### 3.3.4 Dropout reasons and rates

**FIGURE 2:**
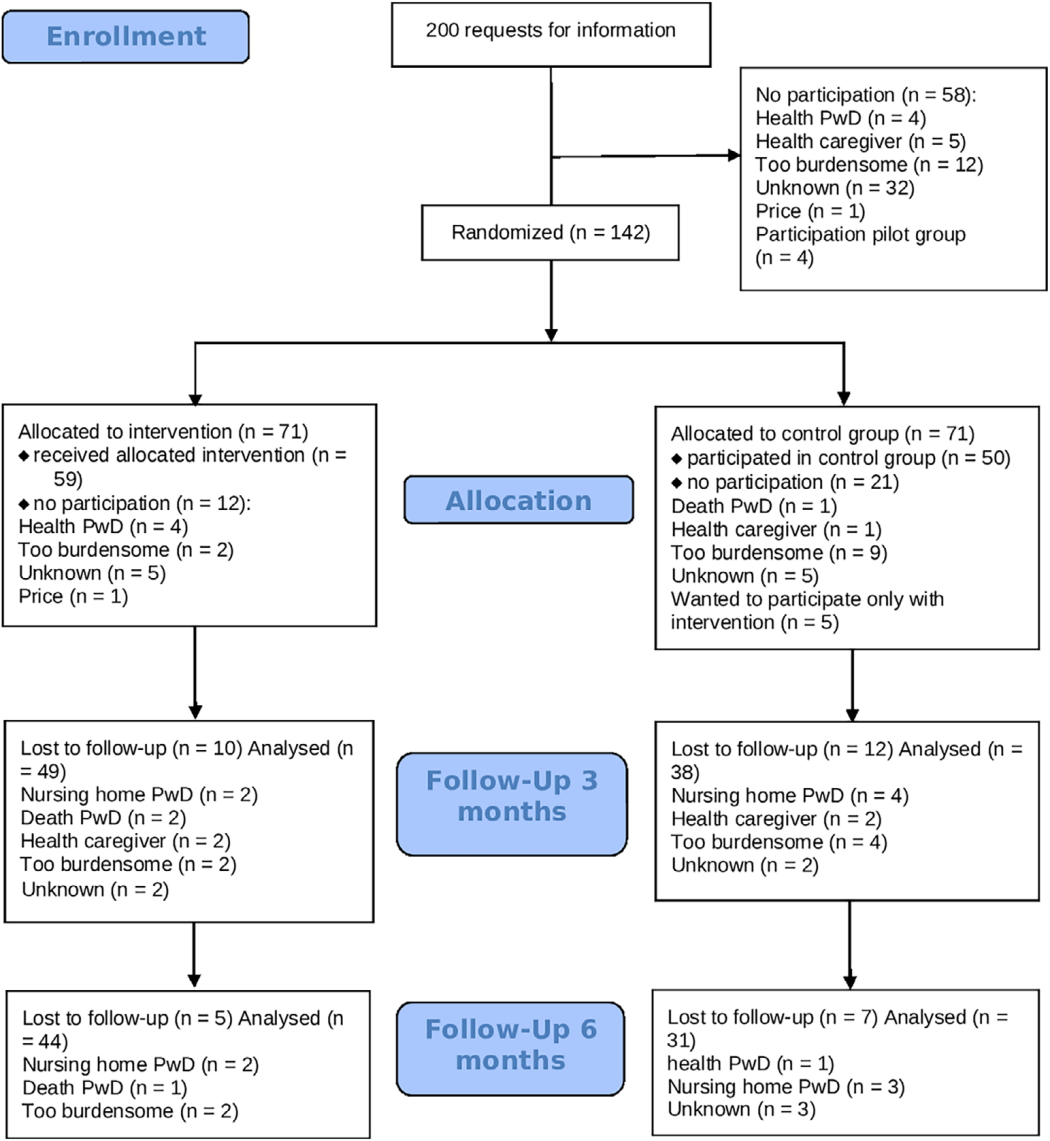
Participation flowchart. Data are for those after request for information and include the reasons for dropout [Colour figure can be viewed at wileyonlinelibrary.com]

Details of the dropouts from allocation to baseline, and during follow‐up, are presented in Figure 2. As stated earlier, no participants dropped out during the intervention. Dropout was because of health (eg, health problems, nursing home admittance and death) for thirteen and twelve couples in the intervention and control groups, respectively. Another common reason for dropout was perceived stress (six in the intervention group and thirteen in the control group); of the six in the intervention group, two stated that this was because completing the questionnaires was time consuming (eg, taking ≥2 hours). In the control group, six caregivers considered the research assistant's visit to be too stressful, two stated that completing the questionnaires was too confrontational, one stated that the questionnaires required too much work and four stated that they had other priorities (eg, work, hobbies or vacations). Five caregivers in the control group opted not to participate because they had only wanted to engage in the intervention week, and another dropped out because of the costs. One caregiver in the intervention group was found to have cognitive problems himself and did not respond to our requests to complete the questionnaires.

#### 3.3.5 Participant characteristics

The external validity of the study was examined by collecting demographic data. The mean age of the caregivers was 72.8 ± 7.6 years (males 76.9 ± 7.4 years, females 71.5 ± 7.3 years); for PWD, the mean age was 76.8 ± 6.9 years (males 77.4 ± 6.9 years, females 75.0 ± 7.4 years). Among PWD, 72% had been educated to the upper secondary level or higher.

## 4 DISCUSSION

In this study, to gain a better understanding of how change occurs, we have described and evaluated the processes involved in delivering an intervention. Exploring the mechanisms through which an intervention leads to change is important when interpreting the quantitative effects of a study and how these can be replicated or augmented in future interventions.^14^ Participants in the present study appeared to benefit from the content of our workshops and from the resulting discussions and time spent together. Modelling (ie, learning from each other's behaviors) was an effective learning tool that helped to augment the self‐esteem of caregivers, while sharing experiences contributed to a group feeling and promoted social support during the intervention, which in some cases, persisted afterward. The importance of being with peers was an important mechanism that we consider essential when considering such interventions for caregivers. Concerning the quantitative effects, the participants indicated that these were probably dispersed over different domains (ie, self‐confidence, knowledge and coping skills), potentially diluting the results. Given the relatively small cohort, this could have led to nonsignificant quantitative outcomes.

Concerning the internal validity, we found that organizing an intervention like “More at Home with Dementia” was feasible in non‐medical settings. Meetings among specialist staff ensured that workshops were standardized, and it was notable that participants adhered to the workshops and completed the intervention. Reasons for nonattendance at workshops were mostly related to health. Community‐based interventions in which people stayed at their own homes have been reported to have discontinuation rates of 10%‐24%.^30^, ^31^, ^32^ Staying at a holiday location may have stimulated workshop attendance and course completion, thereby increasing the chance of the desired effect being achieved. However, our main study results arguably center on issues around external validity.

Our recruitment target was only achieved after extending both the trial period (ie, from 24 to 36 months) and the recruitment activities (ie, from regional to national). Geriatricians and dementia case managers indicated that the main reasons for low referral rates were that patients in their practices were in later stages of dementia, that many had language barriers (eg, a high migration background in Rotterdam) and that the health of caregivers was poor. They also indicated that they were reluctant to recommend the project because half would be assigned to a control group. We could not confirm the proportion of people who were informed about the study but refused to participate, or the reasons for not participating. However, we certainly cannot exclude selection bias by referrers.

Recruitment activities targeting potential participants directly, and publications of detailed personal stories and experiences, were very effective. A similar finding was reported in another study targeting a similar population in the Netherlands.^31^ Thus, we conclude that caregivers of PWD are willing to participate when provided with clear information from reputable sources.

We have continued the intervention without a control group since the study ended and note that participation rates have gradually increased without the need for extensive recruitment activities. This suggests that the randomized controlled design may be an intrinsic barrier to recruitment in such interventions. Indeed, a review of participant recruitment to clinical studies indicated that common reasons for nonentry were preference for one form of treatment, dislike of the idea of randomization and the potential for increased demands.^33^ We also considered the possibility of a waiting group design, as used in the original study of this intervention.^34^ However, this was not possible because we aimed to assess the long‐term effects of the intervention on the time to admittance to a nursing home. It has been possible to continue the intervention because policy makers in Rotterdam participated in the research steering group, and based on the qualitative outcomes, have been willing to finance the intervention.

Few people from non‐Western cultures participated in this study. Although we only included people who could communicate in Dutch, we doubt that this explains the whole difference. Instead, we believe that there may have been cultural differences in accepting dementia as an illness, participating in research and staying at vacation sites with unknown people.

Participants also had a mean age that was approximately 3 years younger than that of PWD in the general population.^35^, ^36^ Moreover, 72% of the PWD were educated to the level of at least upper secondary education, which contrasts with the reported 28% described in an update covering 18 European countries.^35^ Thus, our intervention appeared to be preferentially attended by PWD who were relatively younger and educated to a higher level. Although we did not collect information on the education level of caregivers, we assume that this was comparable. Supporting this, case managers reported that younger and higher educated caregivers were probably more active in seeking help.

We intend to make the intervention more appealing to migrant groups and to people with lower educational levels by organizing an alternative intervention that is more suited to the specific needs of these groups. The main changes will be to have a greater focus on the roles of children and other caregivers through individual and family sessions and to make it possible for this specific group to attend the intervention without needing to stay at a vacation site. It will be interesting to assess whether offering the interventions as a series of workshops in a day care setting lowers the threshold for attending the intervention.

Finally, participants in the control group more frequently reported that completing the questionnaires was too burdensome or that they did not want to participate in the control group (Figure 2). This lends further support to the notion that the randomized control design not only limited recruitment but also limited follow‐up. This appears to be a particular problem when there are marked differences in treatment between the intervention and control groups.^33^ Although we allowed couples from all over the country to participate, we lacked the resources to visit all of them at follow‐up after 3 and 6 months. Unfortunately, it was also difficult for participants to complete all questionnaires, even when supported via telephone, so some questionnaire elements remained incomplete, leading to further data loss and attrition.

### 4.1 Strengths and limitations of the process evaluation

The use of a logbook during the intervention period ensured that we could monitor deviations in the study without recall bias favoring outliers. Thus, we are confident that our data provide a reliable and contemporaneous impression of the intervention's quality and the participants' adherence. Furthermore, we collected information about the motives of many of the caregivers who declined participation or dropped out after randomization. The summaries and recordings of the semi‐structured discussions during meetings served as an invaluable source of information about the impact of the intervention that could not be obtained by questionnaires. Due to the large number of referrers, we could not monitor how many potential participants were given information about the intervention during the study. Instead, we had to rely on anecdotal information provided by some geriatricians and dementia case managers.

### 4.2 Conclusions and implications

Overall, we conclude that the internal validity of the study was high, but that the external validity was unclear. It was especially pleasing that the intervention was delivered as planned, with a low attrition rate and with no dropouts. Given the success of recruitment targeting eligible participants directly, coupled with the improved recruitment since study completion, the intervention may be suitable for many caregivers of PWD. However, more effective recruitment methods will still need to be sought. It also appears that patients do not want to risk being randomized to the control group of a trial, and that this can pose an important barrier to participation. The possible influence of age and education level on the effect of the intervention must also be accounted for in the effect analysis. That way, we can estimate whether the intervention is especially suitable for certain caregiver subgroups (eg, younger and more highly educated) or if it needs adjustment for others (eg, older and less educated). At present, the lack of external validity precludes unconditional extrapolation of the outcomes of the effect analysis to the general population. Regarding the inclusion of more people from non‐Western backgrounds, the intervention and recruitment techniques will need to be adjusted to improve access in future research.

## CONFLICT OF INTEREST

The authors declare that they have no competing interests. However, there were two commercial sources of Funding (THEIA foundation of Zilveren Kruis Health insurance and Laurens Care Centers, Rotterdam, the Netherlands). The commercial funders had no role in the design, conduct, analysis and final publication decisions of the study.

## Data Availability

The data that support the findings of this study are available on request from the corresponding author. The data are not publicly available due to privacy or ethical restrictions.
